# Predicting Abusive Behaviours in Spanish Adolescents’ Relationships: Insights from the Reasoned Action Approach

**DOI:** 10.3390/ijerph19031441

**Published:** 2022-01-27

**Authors:** Ainara Nardi-Rodríguez, María Ángeles Pastor-Mira, Sofía López-Roig, Lidia Pamies-Aubalat, Fermín Martínez-Zaragoza, Victoria A. Ferrer-Pérez

**Affiliations:** 1 Department of Behavioral Sciences and Health, University Miguel Hernandez, 03202 Elche, Spain; mapastor@umh.es (M.Á.P.-M.); slroig@umh.es (S.L.-R.); lpamies@umh.es (L.P.-A.); f.martinez@umh.es (F.M.-Z.); 2 Faculty of Psychology, Balears Island University, 07120 Palma de Mallorca, Spain; victoria.ferrer@uib.es

**Keywords:** abusive behaviours, adolescence, dating violence, prevention, reasoned action approach, evidence-based model

## Abstract

(1) Background: Partner violence prevention programmes do not produce the expected behavioural changes. Accordingly, experts suggest applying evidence-based behavioural models to identify the determinants of abusive behaviours. In this research, we applied the reasoned action approach (RAA) to predict the performance (boys) and acceptance (girls) of abusive behaviours in adolescents. (2) Method: We designed a questionnaire based on the RAA and performed a cross-sectional study. We analysed the predictive capacity of the RAA constructs on intentions with the sample of single adolescents (*n* = 1112). We replicated the analysis only with those who were in a relationship (*n* = 587) and in addition analysed the predictive capacity of intention on future behaviour (3 months later). (3) Results: The hierarchical regression analysis performed with the sample of single adolescents showed that the model explained 56% and 47% of the variance of boys’ intentions to perform the controlling and devaluing behaviours, respectively; and 62% and 33% of girls’ intention to accept them. With those in a relationship, the model explained 60% and 53% of the variance of boys’ intentions to perform the controlling and devaluating behaviour, respectively, and 70% and 38% of girls’ intention to accept them. Intention exerted direct effects on boys’ performance of controlling and devaluing behaviours (31% and 34% of explained variance, respectively) and on girls’ acceptance (30% and 7%, respectively). (4) Conclusions: The RAA seems useful to identify the motivational determinants of abusive behaviours, regardless of adolescents´ relationship status, and for their prediction. Perceived social norms emerge as a relevant predictor on which to intervene to produce behavioural changes with both sexes.

## 1. Introduction

Intimate partner violence (IPV) refers to a spectrum of behaviours, performed by a partner or ex-partner, that causes physical, sexual or psychological harm (WHO). IPV in all its forms has the greatest impact on the health of women and adolescent girls globally, who are also more affected by severe violence and death than men and boys [1,2]. According to the latest macro survey performed in Europe in 2014, one in three women and girls have suffered psychological abuse since the age of 15, and one in five suffered physical and/or sexual violence by a partner [2]. Psychological abuse is a broad concept that includes a wide range of behaviours [3], such as degradation, humiliation, criticizing, insulting, belittling, social isolation; extreme jealously and possessiveness; and, monitoring movements, among others [4]. Similar behaviours are gathered under the well-known Teen Power and Control Wheel [5]. Together with physical and sexual violence, they form pervasive patterns of coercive control [6].

Psychological abuse is the most prevalent form of IPV in adolescence [7,8]. Within psychologically abusive behaviours, controlling and devaluing behaviours are likely to be present at the beginning of an abusive relationship [9]. In Europe, between 52% and 78% of abused females experience both types of abuse [7]. For instance, between 25% and 35% of European girls reported having been constantly checked up on by phone or texts [7]. In Spain, online controlling behaviours and offline devaluing behaviours are experienced by adolescent girls at higher rates (25%) than by women of all ages (9.6%) [10]. Thus, because of their prevalence and likeliness of being present at the beginning of a relationship, these types of behaviours should be a main target for primary prevention programmes.

Evidence on the effectiveness of IPV primary prevention programmes is scarce [11,12]. Whereas a few reviews confirm their effectiveness in preventing IPV in adolescence [11], according to other studies and meta-analyses, only some programmes have been moderately effective at producing cognitive changes (e.g., on sexist attitudes or justification of violence) both immediately and at follow-up, but none achieved sustained behavioural changes [13,14,15]. Experts suggest that the absence of programmes that produce sustained behavioural changes may be due to the lack of theoretical basis on the functional mechanisms that explain abusive behaviours, which is why they recommend targeting individual behavioural changes by designing interventions based on well-established behavioural models [13,14,15].

The Reasoned Action Approach (RAA) [16] is a well-established framework supported by evidence for a wide range of behaviours [17]. It encompasses constructs such as attitudes and beliefs that have been studied in the context of gender based IPV [18], and that are moulded in adolescence [19]. It also includes the perceived norm, which has a great impact on behaviours and is gaining strength in the study of related issues such as dating violence [2,20]. More interestingly, it allows us to study attitude and the perceived norm towards boys and girls respectively performing and accepting specific psychologically abusive behaviours instead of attitudes and the perceived norm towards IPV, as a general and stereotyped concept.

The RAA [16], an evolution of the different theoretical models proposed by the authors [21], states that the main determinant of performing a behaviour is the persons’ intention to perform it (behavioural determinant). In turn, the intention is determined by motivational determinants: the person’s attitude towards him/her performing the behaviour (overall appraisal of their positive and negative consequences of they carrying out the behaviour), the perceived norm (the perception that important others approve of their performing the behaviour and perform the behaviour themselves), and the perceived control (the perception of the behaviour is under their control according to internal and external factors). These three constructs, together with the intention, make up the predictive level of the model. Note that perceived control has to be studied when behaviours are not under volitional control [16]. Hence, in our case, this construct was not considered because violence is instrumental, and thus under volitional control [22]. The explanatory level of the model is constituted by the explanatory beliefs that configure the constructs, which were not analysed in this study.

## 2. Current Research

With the present study we intended to take a first step in the application of an evidence-based model, as recommended by experts [12,14], to assess its utility and identify the determinants of gendered-based abusive behaviours. We applied the RAA to the prediction of a controlling and devaluing behaviour (to *phone or send WhatsApp to my girlfriend to know where she is, who she is with, and when are we going to see each other and to ignore my girlfriend, or punish her with my silence, without giving the reason*). These behaviours were selected based on the results obtained in a previous Delphi Study with experts on gender-based IPV, who considered both relevant for prevention aims [23].

**Figure 1:**
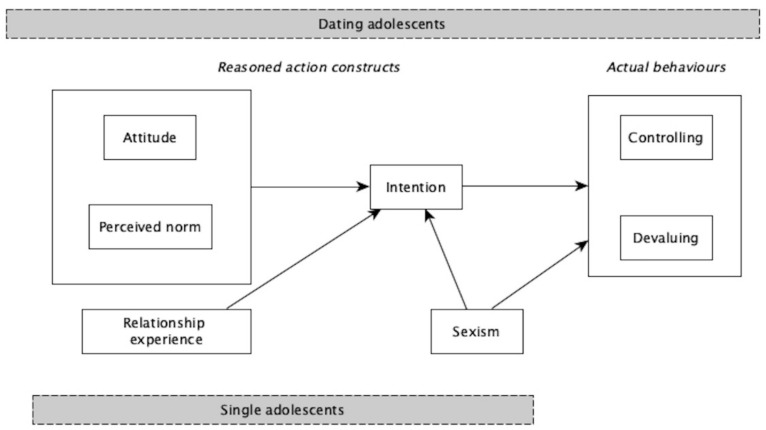
Paths explaining psychological abusive behaviours; Note: in order to simplify the diagram, we indicate the directionality of the relationships among constructs with one arrow.

We designed a cross-sectional study. Our main aims were to: (1) identify the predictors of adolescents’ intention to perform and accept the abusive behaviours and (2) know the capacity of the intention to predict behaviour. Because this second aim could only be analysed with those adolescents that were in a relationship at the time of measuring their performance and acceptance of the behaviours, we tested the whole model with them (sample of dating adolescents). In the case of those that were not in a relationship at that time, we studied the constructs’ predictive capacity for intention (sample of single adolescents) (Figure 1).

We also wanted to know the predictive capacity of sexism on adolescents’ intention to perform and accept the behaviours (sample of single and dating adolescents) and on the prediction of behaviours (sample of dating adolescents). In the study of gender-based IPV, previous research has set the spotlight on sexist attitudes or ambivalent sexism [24,25,26]. However, results regarding their nexus with IPV are inconsistent [27,28]. Some experts consider that this is because attitudes are contingent and contextual, and thus can be triggered during romantic relationships [29]. Hence, we also wanted to know if relationship status was a predictor of boys’ and girls’ respective intention to perform and accept the behaviours (Figure 1).

Previous versions of the RAA have been applied in similar context studies, including to predict cyberbullying [30] and adolescent dating violence [31,32], and have been used as a framework for cyberbullying prevention design [33]. However, in this study we applied the latest version of the Fishbein and Ajzen predictive model [16] from a dyadic perspective; that is, we identified the predictors of the intention to perform and accept the behaviours in a large sample of adolescents with different sociodemographic backgrounds, and the predictive capacity of intention on behaviour.

## 3. Materials and Method

### 3.1. Participants

In total, 1619 heterosexual adolescents aged from 14 to 18 years old participated in the study (*M =* 16). They came from 11 state schools and state supported private schools of a Spanish town. Of these, 1112 teenagers (49.1% boys and 50.9% girls), were not in a relationship (sample of single adolescents) at the time of measuring the performance and acceptance of the behaviours (measure 2). Within these, 164 were at the time of the first measure (to measure the predictors of intention) in a relationship (14.75%) with a duration mean of 6.2 months, 472 had been in a relationship before (42.44%) with a duration mean of 6 months, and 462 had never been in one (41.54%). Three months later we returned to the same schools and asked those adolescents who, during the last 3 months had a relationship of at least one month duration, to answer if they had performed (boys) or accepted (girls) the behaviours (measure 2). A total of 587 adolescents (40.7% boys and 59.3% girls) participated; 115 and 124 boys reported on the performance of the controlling and devaluing behaviour respectively, and 182 and 166 girls on their acceptance.

As stated in the introduction, we focus on boys-to-girls IPV because it strongly impacts on the health of women and adolescent girls. Moreover, according to Spanish Law, gender-based violence is exerted by men and suffered by women, and only takes place in heterosexual relationships [34]. Hence, the questionnaires of those participants who identified as not being heterosexual were withdrawn.

### 3.2. Instruments

In the *Predicting and Changing Behavior* manual developed by Fishbein and Ajzen [16], each construct and their respective items are clearly defined. Therefore, it was only necessary to write down the items referring to the behaviours being studied.

In a previous study, we designed and tested four questionnaires based on Fishbein and Ajzen’s recommendation [16], two regarding the performance and two regarding the acceptance of the controlling and devaluing behaviour, respectively. Items for measuring RAA constructs were identical; only the behaviour under assessment changed (performance of controlling or devaluing behaviour for boys, and accepting control or devaluation for girls). The analysis concluded that good psychometric properties were obtained [35,36], allowing us to use them in the present study. Examples of items are presented below and a sample questionnaire can be found in Appendix A.

#### 3.2.1. Reasoned Action Approach Variables

Each variable was assessed with the average score on the scale used to measure them. The internal consistency of the scales can be found in Table 1.

##### Behavioural Intention

We used four items to assess the intention to perform/accept the behaviour on a 7-point scale. The wording depended on the content of the items (e.g., 1 = totally disagree/7 = totally agree; 1 = totally true/7 = totally false). Example items include *I will phone or send WhatsApp to my girlfriend to know where she is, who she is with*… (Controlling behaviour questionnaire) and *I plan to ignore my girlfriend, or punish her with my silence, without giving the reason* (Devaluing behaviour questionnaire). For girls, the questions were the same regarding accepting receiving phone calls or WhatsApp messages. The same items were presented with the devaluing questionnaire regarding ignoring the girl (boys) or accepting being ignored (girls).

##### Attitude towards Behaviour

We designed a 7-point scale with 12 pairs of dichotomized adjectives to measure the attitude towards performing the corresponding behaviour, taking into account the experiential and instrumental components of this construct [16] (e.g., *for me, calling or sending WhatsApp to my girlfriend to know where she is, who she is with…is*: 1= *not romantic/*7 = *romantic,* 1= *cold/*7 = *passionate, and* 1= *useless* 7 = *useful*). The same dichotomized adjectives were presented in the questionnaire regarding ignoring a girlfriend. For girls, the scale was identical but regarded the acceptance of each behaviour.

##### Perceived Norm

We employed six items, three assessing injunctive norms (e.g., *people that are important to me support me phoning and sending WhatsApp to my girlfriend to know where she is, who she is with* …) and three descriptive norms (*people like me phone or send WhatsApp to their girlfriends to know where they are, who she is with* …), following the RAA authors’ recommendation. Answers to items were given on a 7-point scale and the wording depended on the content of the items (i.e., 1 = disagree/7 = agree; 1 = false/7 = true). We obtained a perceived norm average. Higher scores pointed to a higher perceived social pressure.

##### Sexism

We used the Recio et al. [37] scale for detecting sexism in adolescents, which consists of 26 items measuring hostile (*Women reason worse than men*) and benevolent sexism (e.g., *Women are, by nature, more patient and tolerant than men*) and offers a total mean score on sexism. The answer to items ranged from *totally disagree* (1) to *totally agree* (6).

##### Actual Behaviour

We developed a new scale to measure the performance and acceptance of the behaviours (actual behaviour). We used four items to measure whether participants performed or accepted the behaviours three months after the first evaluation. Answers to items were given on a 7-point scale and the wording depended on the content of the items (e.g., 7 = totally agree/1 = totally disagree; 7 = always/1 = never). For instance, we asked *In the past 3 months I have phoned or sent WhatsApp to my girlfriend to know where is she, who she is with*… or *How frequently have you phoned or sent WhatsApp to your girlfriend to know where is she, who she is with.*

Finally, we asked about participants’ sexual orientation, age, and relationship experience with questions such as whether respondents currently had a partner or had had one before and how long the relationship had lasted.

#### 3.2.2. Procedure

We contacted the directors of 11 secondary schools to explain the project and procedure, highlighting its compliance with the ethical criteria of the university ethics committee and the Helsinki statement. For the selection of the school centres, we used the SPSS Macro RNDSEQ [38] to randomize the 46 secondary school centres listed on a Spanish Autonomous Region website, and contacted the first eleven centres on the list who agreed to participate. A consent report from the adolescents’ legal guardians was a requisite for participation. Questionnaires were self-administered during a normal one-hour class. Within each class, we randomly assigned the questionnaires so that half of the sample answered about the controlling behaviour and the other half answered the questionnaire on devaluing behaviour. Therefore, boys and girls completed questionnaires related to performing or accepting (respectively) a single abusive behaviour. Five hundred and sixty-one adolescents responded to the controlling behaviour questionnaire (279 boys regarding performing the behaviour and 282 girls regarding accepting it) and 551 to the devaluing behaviour questionnaire (267 boys regarding performing the behaviour and 284 girls regarding accepting it).

Three months later, participants that had been in a relationship (of at least 1 month) since the first measure had to report on the performance or acceptance of the behaviour (*actual behaviour*). Questionnaires were coded in order to identify those that belonged to the same participant.

### 3.3. Data Analyses

We used SPSS version 23 for descriptive analyses, the reliability study, and Pearson correlations. We divided the sample into two. With those that were single at the time of measure 2, we analysed the predictions of intention with a hierarchical regression analysis (*n* = 1112). We introduced the RAA constructs (attitude and perceived norm) and, in the second step, sexism and relationship status (grouping actual relationship = 0; previous relationship = 1).

For those who were in a relationship three months later (sample of dating adolescents), we conducted a path analysis to analyse the entire model considering the prediction of the reported behaviours in time 2 (*n* = 587). The hypothesized paths are depicted in Figure 1. The path analysis was performed by structural equation modelling (SEM) using the lavaan package [39] of the R Statistical Package [40]. Mardia’s multivariate normality test and Kolmogorov–Smirnov univariate normality tests were undertaken using the MVN package in R [41], and showed a non-normal data distribution. Therefore, we used a Satorra–Bentler scale (mean adjusted) test statistic for our estimation method.

A fit criteria assessment was conducted according to the Hu and Bentler study [42]. The goodness-of-fit statistical test assesses the magnitude of unexplained variance. A ratio of χ^2^/gL < 2 suggests an acceptable fit. The chi-square statistic provides a conventional measure of model fit. However, because of its sensitivity to sample size, two additional fit indices were used to supplement the chi-square statistic. The choice of these two indices was based on Hu and Bentler’s recommendation [43] of a two-index presentation strategy, which was found to provide an optimal balance between Type I and Type II error rates. A RMSEA size below 0.06 suggests a well-fitting model. A CFI above 0.95 indicates a good fit. A SRMR of less than 0.09 also indicates a good fit.

## 4. Results

### 4.1. Sample of Single Adolescents

Correlational analysis showed significant relationships between the different constructs of the RAA model. All variables were significantly correlated with the boys’ intention of controlling behaviour (except for attitude) and devaluing behaviours (except for previous relationship), and with girls’ intention of accepting the controlling behaviour (except for previous and current relationship) and the devaluing behaviour (except for actual relationship) (Table S1).

For the boys, regression analyses showed that perceived norm was the only significant predictor of the intention to control their partner and accounted for 55% of the explained variance of the intention. In the case of the devaluing behaviour, attitude, perceived norm, and sexism accounted for 47% of the explained variance of the intention of performing the behaviour. Perceived norm had the greatest weight as predictor of the intention (Table 2). For the girls, attitude and perceived norm were significant predictors of the intention of accepting both the controlling behaviour (62%) and the devaluing behaviour (33%).

Sexism was only a significant predictor in the case of the boys’ intention to perform the devaluing behaviour (*B* = 0.14 [0.04, 0.24]) but not for the controlling behaviour (*B* = 0.12 [−0.03, 0.26]). For the girls, sexism was not a significant predictor of the intention to accept any of the behaviours; nor was relationship experience (previous or current) a predictor of boys’ or girls’ intentions (Table 2).

### 4.2. Sample of Adolescents in a Dating Relationship

Results of the correlational analysis showed, for the boys, that attitude and perceived norm significantly correlated with the devaluing intention measure. For the controlling intention measure, only perceived norm correlated. In the case of the girls, attitude and perceived norm correlated with the intention to accept the controlling and devaluing behaviour. Intention was significantly correlated to actual behaviour for all the behaviours in both sexes, ranging from *r* = 0.27 (*p* ≤ 0.05) for accepting the devaluing behaviour to *r* = 0.56 (*p* ≤ 0.001) for performing the controlling behaviour (Table S2).

In Table 3, we present the fit indices associated with the initial and final four models. All indices indicated a good fit, except χ^2^/df and RMSEA for the initial model of devaluing behaviour in girls. Deleting the non-significant paths, all fit indices improved in the latter case, indicating that this final model fits the data within the established fit criteria.

The standardized beta parameters for the first SEM structural analyses were calculated (Table S3).

**Figure 2:**
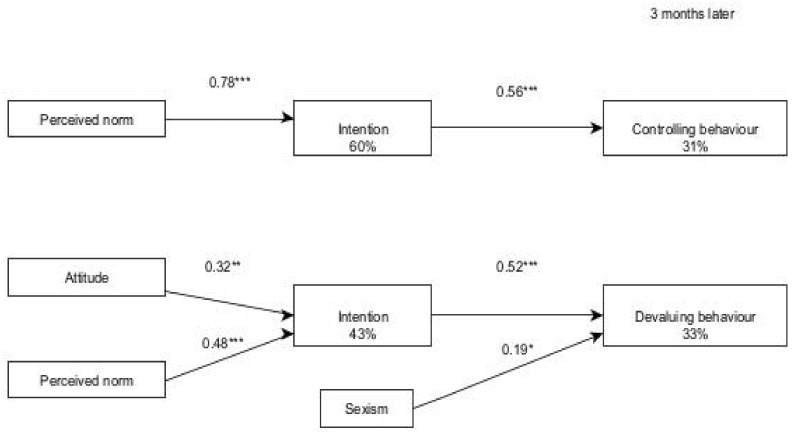
Final models for performing abusive behaviours among boys in a relationship (three months after). Notes: * *p* < 0.05. ** *p* < 0.001. *** *p* < 0.001.

In order to represent the results graphically, we present the final models with significant standardized β parameters. In the boys, perceived norm had a direct effect on the intention to control a partner with 60% of explained variance, and together with attitude they accounted for 34% of the intention to devalue a partner. Intention had a direct effect on both behaviours with very similar explained variance (Figure 2).

**Figure 3:**
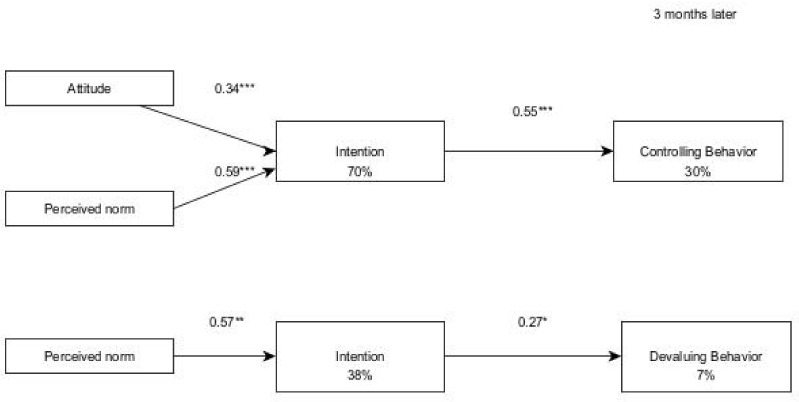
Final models for accepting abusive behaviours among girls in a relationship (three months after). Notes: * *p* < 0.05. ** *p* < 0.001. *** *p* < 0.001.

For the girls, attitude and perceived norm had direct effects on the intention to accept being controlled by their partners, with 70% of explained variance, and perceived norm by itself explained 38% of the intention to accept the devaluing. Intention had a direct effect on accepting controlling and devaluing behaviours three months later (30% and 7% of explained variance, respectively). In addition, sexism had only a small direct effect on the performance of the devaluing behaviour 3 months later (β = 0.19; *p* ≤ 0.05), but no effect on the performance and acceptance of the controlling behaviour or acceptance of the devaluing behaviour (Figure 3).

## 5. Discussion

The study aims were to identify the motivational determinants of boys’ and girls’ intentions to perform and accept respectively the behaviours, to explore the contribution of sexism and relationship experience to the prediction of intention (sample of single and dating adolescents), and identify intention´s predictive capacity for behaviours 3 months later (sample of dating adolescents).

The RAA is a useful tool for the prediction of these abusive behaviours, as shown in a previous study [32]. Attitude and perceived norm explained considerable proportions of the variance of the intention to perform and accept the behaviours among the sample of adolescents that were single and among those in a relationship over a 3-month period. The explained variance of intentions and behaviours exceeds the percentage of explained variance found by Armitage and Conner [17] in their review, with the exception of accepting the devaluating behaviour, for which the explained variance was low. This may be due to the limited variability of its acceptance by adolescent girls in a relationship in the second measurement.

Surprisingly, contrary to the relevance given to attitudes in the study of gender-based IPV [18], we must point out that attitudes towards performing and accepting a behaviour do not always act as predictors. Only the perceived norm played a role as a predictor of the intention to perform (boys) and accept (girls) the behaviours in both samples. In this regard, Reed et al. [2] found that adolescents who admitted exerting any form of gender-based partner violence perceived that their friends did the same. Taylor’s et al. [44] findings also support the importance of friends’ norms as a predictor of adolescents’ perpetration of physical dating violence. Other studies have found a link between peer norms and antisocial behaviours [45].

Another issue to underline is that predictors varied according to the behaviour. For instance, whereas perceived norm was the only predictor of the intention to perform the controlling behaviour and its performance in both samples, perceived norm and attitude were both predictors of the intention and acceptance of this same behaviour. This suggests that prevention strategies should be different in boys and girls. This differs from standard practice in Spain, where boys and girls work together in prevention programmes, as deduced in an in-depth review of prevention programmes [46].

Concerning the role of sexism, contrary to our expectations, results suggest that it does not play a key role as a predictor of intentions and of the performance and acceptance of the controlling behaviour. Only in the case of intending to perform the devaluing behaviour and its performance over a 3-month period, did sexism act as predictor. However, its effect was small. Therefore, this result suggests that intervention regarding sexist attitudes may not produce specific behavioural changes. This may reflect the need to develop new instruments, such as implicit attitude scales, to measure this construct accurately with youth who are aware that sexism is a socially rejected issue. Conversely, it is possible that sexism is not predictive because we are all socialized in sexist cultures. Recent studies also found that sexism is not a good predictor [47]. The different levels of generality (sexism vs. specific behaviour) may be another possible explanation for our results, in line with the RAA. In other words, values or general beliefs about women or girls may differ from values and beliefs that support specific abusive behaviours. Nonetheless, although sexism does not exert a direct influence on intentions and behaviours, it seems to have an effect through the configuration of the explanatory beliefs that underlie the performance and acceptance of these behaviours identified in previous studies [34,35], as the authors of the model state [16].

With respect to relationship experiences, results showed that they were not a predictor of the intention to perform and accept the behaviours among adolescents. That is, having a relationship or having had one before does not influence the intention to perform and accept the behaviours. This is contrary to our expectations, because the study of Arriaga et al. [29] found that, among adults, being in a relationship decreased people’s tolerance to abusive behaviours. Nevertheless, our findings indicate that an identical prevention programme for these behaviours can be applied to adolescents regardless of their relationship experiences, which facilitates their implementation.

This study has some limitations. First, we worked with a school population and thus results are only generalizable to this population. According to a previous study [48], a considerable proportion of aggressors undertook secondary or university studies. Second, socioeconomic background information of the sample was not required. However, the schools belonged to different socioeconomic level neighbourhoods, thus ensuring the representativeness the sample. Third, the results only apply to heterosexual and male perpetrators of partner violence, and thus cannot be extended to other forms of partner violence. Fourth, we worked with self-reported data, and thus participants may have under-reported the performance and acceptance of abusive behaviour. Finally, the correlational nature of the study should be considered and, as a result, we have not identified causal factors but predictors.

## 6. Conclusions

The RAA [16] is a good model for the prediction of the controlling and devaluing behaviours under study. Our results suggest three main practical and research implications: First, to refine intervention effectiveness (and time-cost effectiveness), school-based prevention programmes may benefit from intervening in behavioural determinants instead of systematically intervening in general attitudes. The second implication is that to prevent this issue effectively, programmes may benefit by intervening differently in boys and girls because the determinants of performing and accepting the same behaviours vary, in addition to the explanatory beliefs [34,35]. The third implication is that perceived norm seems to be an important variable, and thus the variable should be incorporated as an individual aim in those prevention programmes that tend to focus mainly on attitudinal aspects. Finally, results also suggest that continuing to apply the model to other psychologically abusive behaviours would increase the probability of developing an evidence-based prevention programme as recommended by experts.

## Figures and Tables

**Table 1:** Internal consistency of boys’ and girls´ subscales for controlling and devaluing behaviours.

	Single Adolescents				Dating Adolescents			
	Boys		Girls		Boys		Girls	
	CB	DB	CB	DB	CB	DB	CB	DB
Behavioural intention	0.94	0.91	0.95	0.90	0.96	0.92	0.95	0.90
Attitude	0.93	0.91	0.93	0.87	0.94	0.89	0.94	0.85
Perceived norm	0.86	0.75	0.82	0.71	0.83	0.74	0.83	0.76
Sexism	0.94	0.94	0.92	0.93	0.94	0.94	0.90	0.94
Actual Behaviour	-		-		0.91	0.94	0.94	0.96

Note. CB = Controlling behaviour; DB = Devaluing behaviour.

**Table 2:** Predictive models of intention to perform and accept the behaviours in the study with single adolescents.

Boys						
Criterion: Intention ^†^	Predictors	R^2^	F	df	β	B[CI]
Controlling behaviour		0.56	62.40 ***	3239		
	Attitude				0.02	0.74 [−0.26, 0.41]
	Perceived norm				**0.71**	0.85 [0.75, 0.96]
	Sexism				0.08	0.15 [−0.03, 0.31]
	Previous relationship				0.04	0.14 [−0.13, 0.41]
	Actual relationship				0.03	0.30 [−0.42, 1.03]
Devaluing behaviour		0.47	40.19 ***	3216		
	Attitudes				**0.26**	0.24 [0.14, 0.35]
	Perceived norm				**0.51**	0.57 [0.45, 0.68]
	Sexism				**0.13**	0.14 [0.03, 0.25]
	Previous relationship				0.03	0.06 [−0.14, 0.28]
	Actual relationship				0.03	0.16 [−0.44, 0.77]
**Girls**						
Controlling behaviour		0.62	79.72 ***	3238		
	Attitude				**0.48**	0.54 [0.44, 0.64]
	Perceived norm				**0.44**	0.51 [0.41, 0.61]
	Sexism				0.05	0.10 [−0.04, 0.24]
	Previous relationship				0.04	0.11 [−0.09, 0.32]
	Actual relationship				0.03	0.29 [−0.33, 0.91]
Devaluing behaviour		0.33	23.72 ***	2231		
	Attitude				**0.22**	0.25 [0.13, 0.39]
	Perceived norm				**0.49**	0.58 [0.45, 0.71]
	Sexism				0.01	0.01 [−0.12, 0.15]
	Previous relationship				0.05	0.12 [−0.1, 0.34]
	Actual relationship				−0.02	−0.23 [−1.2, 0.74]

Notes. ^†^ =Reported the last model; bold*=* significant predictor; ***: *p* ≤ 0.001.

**Table 3:** Fit indices of the initial and final models in the study with adolescents in dating relationships.

Initial Models				
Fit Indices	Boys		Girls	
	Controlling	Devaluing	Controlling	Devaluing
χ^2^/df ^†^	0.75 ns	0.14 ns	2.21 ns	4.45 ns
CFI	1.00	1.00	0.99	0.97
RMSEA	0.00 [0.00, 0.14] ^b^	0.00 [0.00, 0.05]	0.02 [0.00, 0.17]	0.09 [0.00, 0.20]
SRMR	0.01	0.01	0.01	0.03
Final models				
χ^2^/df	0.24 ns	0.55 ns	2.21 ns	1.17 ns
CFI	1.00	1.00	0.99	0.98
RMSEA	0.00 [0.00, 0.19]	0.00 [0.00, 0.00]	0.03 [0.00, 0.16]	0.03 [0.00, 0.00]
SRMR	0.01	0.02	0.02	0.02

Notes. ^†^: df_initial models_ = 2; df_final models_: Controlling behaviour = 1 for boys and 2 for girls; Devaluing behaviour = 3 for boys and 2 for girls; ^b^: 90% CI; ns = no significative.

## Data Availability

The data presented in this study are available on request from the corresponding author.

## Supplementary Materials

The following are available online at https://www.mdpi.com/article/10.3390/ijerph19031441/s1, Table S1: Descriptive statistics and correlations in the study with single adolescents, Table S2: Descriptive statistics and correlations in the study with adolescents in dating relationships, Table S3: Standardized β parameters for the first models in the study with adolescents in dating relationships.

## Appendix A. Questionnaire Model

Below a sample questionnaire can be found with regard to performing the controlling behavior understudy. For the rest of behaviors, the questionnaire was identical. Only the behavior under study changed.

Note that the original questionnaire was wrote in Spanish. Previous to its’ use, it is necessary to perform a professional translation and transformation by means of a comprehensive psychometric.

In the questionnaire we indicate the construct to which each item belongs.

- Intention scale items: INT 1+ INT 2+ INT 3+ INT 4
  ○ Subjective norm scale items:
  ○ Prescriptive items: SNP1+SNP2+SNP3
  ○ Descriptive items: SND1+SND2+SND3
- Attitude scale items:
  ○ Instrumental items: 2, 5, 6, 7, 11, 12
  ○ Experiential items: 1, 3, 4, 8, 9, 10

### Instructions

This questionnaire is about how we relate to our partners. To answer, you must circle the number from 1 to 7 that indicates your opinion on the corresponding scale similar to the following one:

**1**	**2**	**3**	**4**	**5**	**6**	**7**
Totally agree	Strongly agree	Slightly agree	Indifferent	Slightly disagree	Strongly disagree	Totally disagree

The meaning of the numbers will always be the same, but be careful when answering because the adjectives at the ends will change.

Before answering, carefully read the situation that we present to you below. As you know, there are many ways to relate when you go out with someone. The questions that we ask you have to do with the situation that we describe in the box. It is important that you keep it in mind when responding.

When we have been dating a girl for a while, we usually meet, have a drink, go out to a party, go to the movies or be with friends. But it is not always like that! We can stay separately with friends or do some activity on our own. Whenever this happens, some of us have the habit of frequently calling our girls to find out where they are, who they are with or when we are going to see each other. The following questions are about this and we would like to know your opinion. There are no good or bad answers, all opinions are equally valid.

Please read each question carefully. Although some may seem very similar to you, they are not identical and it is important that you answer to all of them. Remember, we are interested in knowing what you think about calling or sending WhatsApp to your girl to find out where she is, with whom, what she does or when you will see each other, when you stay separately with friends or do some activity on your own.

If you’re not currently dating a girl, imagine what you would actually do if you were.

SNP1: The majority of people who are important to me think I should phone or send WhatsApps to my girlfriend to know where she is, who she is with, and when are we going to see each other.

**1**	**2**	**3**	**4**	**5**	**6**	**7**
Totally agree	Strongly agree	Slightly agree	Indifferent	Slightly disagree	Strongly disagree	Totally disagree

INT1: I intend to phone or send WhatsApps to my girlfriend to know where she is, who she is with, and when are we going to see each other.

**1**	**2**	**3**	**4**	**5**	**6**	**7**
Totally agree	Strongly agree	Slightly agree	Indifferent	Slightly disagree	Strongly disagree	Totally disagree

SND1: Most people like me would phone or send WhatsApps to their girlfriends to know where they are, who they are with, and when are they going to see each other.

**1**	**2**	**3**	**4**	**5**	**6**	**7**
Totally agree	Strongly agree	Slightly agree	Indifferent	Slightly disagree	Strongly disagree	Totally disagree

INT2: I plan to phone or send WhatsApps to my girlfriend to know where she is, who she is with, and when are we going to see each other.

**1**	**2**	**3**	**4**	**5**	**6**	**7**
Totally true	Quite true	Slightly true	Indifferent	Slightly false	Quite false	Totally false

SND2: Most people important to me phone or send WhatsApps to their girlfriends to know where they are, who they are with, and when are they going to see each other.

**1**	**2**	**3**	**4**	**5**	**6**	**7**
Totally agree	Strongly agree	Slightly agree	Indifferent	Slightly disagree	Strongly disagree	Totally disagree

INT 3: I have the intention to phone or send WhatsApps to my girlfriend to know where she is, who she is with, and when are we going to see each other

**1**	**2**	**3**	**4**	**5**	**6**	**7**
Totally agree	Strongly agree	Slightly agree	Indifferent	Slightly disagree	Strongly disagree	Totally disagree

SND3: Most people like me would phone or send WhatsApps to their girlfriends to know where they are, who they are with, and when are they going to see each other

**1**	**2**	**3**	**4**	**5**	**6**	**7**
Totally unlikely	Strongly unlikely	Slightly unlikely	Indifferent	Slightly likely	Strongly likely	Totally likely

SNP2: It is expected from me that I phone or send WhatsApps to my girlfriend to know where she is, who she is with, and when are we going to see each other

**1**	**2**	**3**	**4**	**5**	**6**	**7**
Totally true	Quite true	Slightly true	Indifferent	Slightly false	Quite false	Totally false

INT4: I will phone or send WhatsApps to my girlfriend to know where she is, who she is with, and when are we going to see each other.

**1**	**2**	**3**	**4**	**5**	**6**	**7**
Totally unlikely	Strongly unlikely	Slightly unlikely	Indifferent	Slightly likely	Strongly likely	Totally likely

SNP3: Most people who are important to me support me phoning or sending WhatsApps to my girlfriend to know where she is, who she is with, and when are we going to see each other.

**1**	**2**	**3**	**4**	**5**	**6**	**7**
Totally agree	Strongly agree	Slightly agree	Indifferent	Slightly disagree	Strongly disagree	Totally disagree

In the following table, you will find different bipolar adjectives (e.g., totally useful/ totally useless; totally beneficial/totally detrimental). Please select the one which adjusts more to your opinion in regards to phoning or sending WhatsApp to your girlfriend to know where she is, who she is with, and when are we going to see each other.

In your opinion, phoning or sending WhatsApps to your girlfriend to know where she is, who she is with, and when are we going to see each other, is:

	**Totally**	**Quite**	**Slightly**	**Indifferent**	**Slightly**	**Quite**	**Totally**	
1. Romantic	1	2	3	4	5	6	7	Non-romantic
2. Necessary	1	2	3	4	5	6	7	Unnecessary
3. Funny	1	2	3	4	5	6	7	Boring
4. Tough	1	2	3	4	5	6	7	Tender
5. Bad	1	2	3	4	5	6	7	Good
6. Useless	1	2	3	4	5	6	7	Useful
7. Helpful	1	2	3	4	5	6	7	Harmful
8. Stressing	1	2	3	4	5	6	7	Relaxing
9. Passionate	1	2	3	4	5	6	7	Cold
10. Unpleasant	1	2	3	4	5	6	7	Pleasant
11. Intelligent	1	2	3	4	5	6	7	Stupid
12. Protective	1	2	3	4	5	6	7	Attacking
