# Supplementary material for: Predicting Abusive Behaviours in Spanish Adolescents’ Relationships: Insights from the Reasoned Action Approach
Source: Int J Environ Res Public Health. 2022 Jan 27;19(3):1441. doi: 10.3390/ijerph19031441 (PMC8834858; doi:10.3390/ijerph19031441)
Supplement: Supplementary file 1 [file ijerph-19-01441-s001.zip › ijerph-1540707-supplementary.pdf]

International Journal of
Environmental Research and Public Health

MDPI

Supplementary Materials

Table S1. Descriptive statistics and correlations in the study with single adolescents.

	BOYS					GIRLS				
Controlling behavior	M± SD	1	2	3	4	M± SD	1	2	3	4
1. Intention	2.95±1.57		0.81	**0.74**	**0.30**	2.22±1.33		**0.68**	**0.68**	**0.31**
2. Attitude	3.96±0.40			0.09	0.05	2.78±1.19			**0.44**	**0.27**
3. Perceived norm	3.50±1.30				**0.29**	3.12±1.14				**0.21**
4. Sexism	2.18±0.88					1.84±0.74				
	n (%)									
Previous relationship†	148 (60.2)					116 (47%)				
Actual relationship‡	38 (13.7)					40 (14.3%)				
Devaluing behavior	M± SD	1	2	3	4	M± SD	1	2	3	4
1. Intention	1.80±1.08		**0.46**	**0.62**	**0.30**	1.57±1.03		**0.32**	**0.58**	0.14 *
2. Attitude	2.19±1.12			**0.33**	**0.27**	1.79±0.87			**0.19**	**0.17**
3. Perceived norm	2.54±0.97				**0.20**	2.30±0.87				**0.17**
4. Sexism	2.20±0.98					1.84±0.80				
	n (%)									
Previous relationship	111 (48.5)					117 (48.5)				
Actual relationship	40 (15.2)					46 (16.2)				

Note. *: $p \leq .05$; bold= $p \leq .01$; Boys were assessed regarding the performance of the behavior and girls regarding its acceptance. †Previous relationship: For controlling behavior N_{boys} = 246 and N_{girls} = 247; For devaluing behavior N_{boys} = 229 and N_{girls} = 241. ‡Actual relationship: for controlling behavior N_{boys} = 278 and N_{girls} = 280; For devaluing behavior N_{boys} = 264 and N_{girls} = 284.

Table S2. Descriptive statistics and correlations in the study with adolescents in dating relationships.

	BOYS					GIRLS				
Controlling behavior	M± SD	2	3	4	5	M± SD	2	3	4	5
1.Actual behavior	3.64±1.65	**0.56**	0.13	**0.41**	0.21 *	3.57±1.82	**0.54**	**0.44**	**0.47**	0.12
2. Intention	3.18±1.68		0.15	**0.78**	**0.29**	2.81±1.73		**0.70**	**0.80**	0.17 *
3. Attitude	4.02±0.55			0.18	0.17	2.98±1.25			**0.61**	**0.29**
4. Perceived norm	3.68±1.24				**0.35**	3.54±1.29				**0.22**
5. Sexism	2.09±0.84					1.77±0.64				
Devaluing behavior	M± SD	2	3	4	5	M± SD	2	3	4	5
1.Actual behavior	2.14±1.51	**0.55**	**0.31**	**0.32**	**0.29**	1.79±1.33	**0.27**	−0.03	**0.22**	0.11
2. Intention	1.88±1.28		**0.49**	**0.59**	0.19 *	1.52±0.95		**0.31**	**0.57**	**0.31**
3. Attitude	2.09±0.84			**0.36**	0.17	1.57±0.72			0.18 *	0.18 *
4. Perceived norm	2.75±0.99				0.16	2.34±1.00				0.22 *
5. Sexism	2.08±0.82					1.77±0.76				

Note. *: $p \leq .05$; bold= $p \leq .01$; Boys were assessed regarding the performance of the behavior and girls regarding its acceptance.

Table S3. Standardized β parameters for the first models in the study with adolescents in dating relationships.

	BOYS			
	Intention †	Controlling	Intention ‡	Devaluing
Attitude	0.00		0.31 **	
Perceived norm	0.77 ***		0.47 ***	
Sexism	0.01	0.06	0.06	0.19 *
Intention		0.53 ***		0.52 ***
	GIRLS			
	Intention †	Controlling	Intention ‡	Devaluing
Attitude	0.36 ***		0.19	
Perceived norm	0.59 ***		0.51 **	
Sexism	−0.08	0.04	0.16	0.01
Intention		0.55 ***		0.27 **

Notes. *Intention*† = Intention of controlling; *Intention*‡ = Intention of devaluing; *: $p \leq .05$; **: $p \leq .01$; ***: $p \leq .001$; Note: boys were assessed regarding the performance of the behavior and girls regarding its acceptance.
